# Hegemann’s disease and fishtail deformity: aetiopathogenesis, radiographic appearance and clinical outcome

**DOI:** 10.1007/s11832-014-0630-z

**Published:** 2015-01-11

**Authors:** Femke M. A. P. Claessen, Jan K. G. Louwerens, Job N. Doornberg, C. Niek van Dijk, Michel P. J. van den Bekerom, Denise Eygendaal

**Affiliations:** 1 Orthopaedic Hand and Upper Extremity Service, Massachusetts General Hospital, Harvard Medical School and University of Amsterdam Medical School, Boston, MA USA; 2 VU Medical Center, Orthopaedic Residency Program (PGY1), Amsterdam, The Netherlands; 3 Orthotrauma Research Center Amsterdam, University of Amsterdam Orthopaedic Residency Program (PGY4), Amsterdam, The Netherlands; 4 Department of Orthopaedic Surgery, Academic Medical Center Amsterdam, Amsterdam, The Netherlands; 5 Shoulder and Elbow Unit, Onze Lieve Vrouwe Gasthuis, Amsterdam, The Netherlands; 6 Upper Limb Unit, Amphia Hospital, Breda, The Netherlands

**Keywords:** Osteochondrosis, Fishtail deformity, Hegemann’s disease, Children, Distal humerus pain, Elbow injury

## Abstract

**Purpose:**

A systematic review regarding clinical studies on Hegemann’s disease and fishtail deformity was performed with the aims to: (1) formulate the most up-to-date theory on aetiology in order to better define these conditions, (2) summarise the most frequent radiographic descriptions on radiographs and (3) give an overview of different treatment options.

**Methods:**

A systematic review of studies to date on Hegemann’s disease and fishtail deformity was performed. Studies were eligible if: (1) the article provides a description of Hegemann’s disease or fishtail deformity, (2) original data of at least one patient was available, (3) the article was written in English, German or Dutch and (4) a full manuscript was available. Animal studies, reviews and expert opinions were not included.

**Results:**

We included a total of 22 articles: seven regarding Hegemann’s disease including eight patients and 15 regarding fishtail deformity including 58 patients.

**Conclusions:**

Fishtail deformity and Hegemann’s disease seem to be a spectrum of vascular disorders of the distal humerus, varying from a benign mild vascular disorder to a complete avascular necrosis after fractures. Additional imaging is advised to confirm the presence of a fishtail deformity, intra-articular loose bodies and signs of osteoarthritis to decide if, and what, operative treatment is needed. As long as no clear aetiology for both diseases exist and the clinical symptoms and radiographic appearance are difficult to distinguish, both entities should preferably be named as ‘vascular disturbance of the trochlear growth plate’ to overcome confusing definitions and discussions.

## Introduction

Osteochondrosis is used to describe more than 50 different conditions affecting the immature skeleton [1]. In 1951, Dr. Gerd Hegemann described the radiographic changes of the humeral trochlea in the young adult; therefore, osteochondrosis of the humeral trochlea is known as Hegemann’s disease [2]. Studies so far report that osteochondrosis goes through stages, similar to Perthes' disease [3]. Reports on patients with Hegemann’s disease are very rare [2]. Fishtail deformity of the elbow is a rare complication after fracture of the distal humerus, usually following a supracondylar, a lateral condyle, a medial condyle or even after Salter–Harris type I epiphyseal fractures in childhood [4].

Aetiology as well as optimal treatment for Hegemann’s disease and fishtail deformity are subjects of ongoing debate. A systematic review regarding clinical studies on Hegemann’s disease and fishtail deformity was performed with the aims to: (1) formulate the most up-to-date theory on aetiology in order to better define these conditions, (2) summarise the most frequent radiographic descriptions on radiographs and (3) give an overview of different treatment options.

## Materials and methods

### Search strategy

To identify studies focusing on Hegemann’s disease and fishtail deformity, the following databases (up to 27th August 2014) were searched: EMBASE, MEDLINE OvidSP, Web of Science, Cochrane Central, PubMed Publisher, Scopus and Google Scholar (Table 1). The EMBASE search strategy was transferred into similar search strategies for the other databases. References of the included articles were also searched to identify more potentially relevant literature.

**Table 1:**
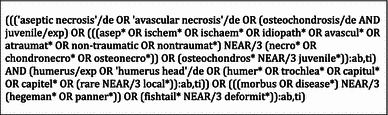
Search strategy

### Study selection

Study selection was assessed by two independent reviewers (FC and JL). Disagreements were solved by consensus. If no consensus was reached, a third reviewer (MB) solved the disagreement. Studies were eligible if: (1) the article provides a description of Hegemann’s disease and/or fishtail deformity, (2) original data of at least one patient were available, (3) the article was written in English, German or Dutch and (4) a full-text article was available. Animal studies, (systematic) reviews and expert opinions were not included.

### Methodological quality assessment

Two reviewers (FC and JL) independently assessed the methodological quality of all the included studies. Important aspects of methodology were noted: study design, follow-up time and outcomes, e.g. because all studies were case reports, no pre-printed selection forms or an overall scoring system to evaluate methodological quality was used [5].

### Data extraction

Data extraction was performed by the first author (FC). The following data were extracted: study population, patient characteristics, design of study, aetiology, clinical presentation and physical examination, radiological evaluation, treatment and outcome measures.

## Results

### Literature search

**Fig. 1:**
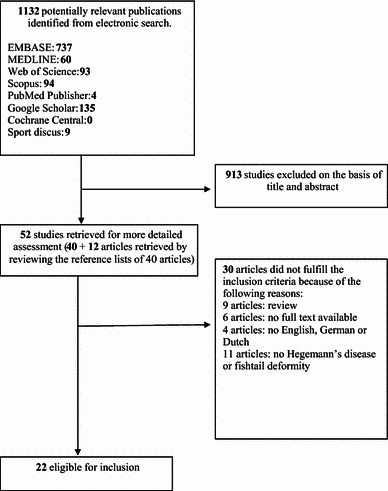
Flow diagram of the study selection and exclusion stages

A total of 22 studies were included in the current review (Fig. 1), comprising seven studies regarding Hegemann’s disease and 15 studies regarding fishtail deformity.

### Hegemann’s disease

Seven case reports regarding Hegemann’s disease, including eight patients, were analysed [1, 2, 6–10]. The study and patient characteristics are shown in Table 2.

**Table 2:** Hegemann’s disease—patients and study characteristics

Author(s), year, country	Study type	No. of males (%)	Age, mean (years)	Follow-up (months)	Sports	Aetiology	Symptoms	Imaging	Treatment	Outcome parameters
Hegemann, 1951, Germany [2]	Case report	1 (100)	13	16	–	Elbow contusion	Pain, swollen elbow, limited ROM	X-ray: irregularity trochlea	Conservative	Clinical symptoms, elbow function, X-ray
Szepesi, 1971, Hungary [9]	Case report	1 (100)	15	12	–	Atraumatic	Pain, limited ROM	X-ray: irregularity trochlea and epiphysis. Sclerotic trochlea. Fragmented trochlea	Conservative, rest, less strain to the elbow	Clinical symptoms, elbow function, X-ray
Mueller and Haehnel, 1976, Germany [8]	Case report	1 (0)	13	–	Gymnastics	Sports	Limited ROM	X-ray: irregularity trochlea	–	–
Osebold et al., 1977, USA [10]	Case report	1 (100)	8	18	–	–	Limited ROM	X-ray: flattening of the trochlear ossific nucleus. Sclerosis trochlea. Aseptic necrosis trochlea	–	X-ray
Martin and Ehrenpfordt, 1984, Germany [7]	Case reports	2 (100)	12	48	–	Case 1: elbow contusion	Pain, swollen elbow, limited ROM	X-ray: irregularity trochlea	Conservative, rest, less strain to the elbow	Clinical symptoms, X-ray
						Case 2: fracture in past history		Smaller surface trochlea		
Beyer et al., 1990, Germany [1]	Case report	1 (100)	7	36	–	Fracture in past history	Limited ROM	X-ray: osteolysis trochlea	–	X-ray
Ito et al., 2004, Japan [6]	Case report	1 (100)	11	60	–	–	Pain, swollen elbow, limited ROM	X-ray: sclerosis with partial defect and irregular contour of the humeral trochlea epiphysis	Conservative, less strain to the elbow	Clinical symptoms, elbow function, X-ray

*ROM* range of motion

#### Aetiology

In six case reports, the aetiology of Hegemann’s disease was described [1, 2, 6–9]. Two cases reported contusion of the elbow (25 %) [2, 7] and in two patients, a fracture in the past history was described (25 %) [1, 7]. In two patients, no trauma were reported in the history (25 %) [9, 10] and in one case, Hegemann’s disease presented in a gymnast (13 %) [8].

#### Patient characteristics

In all eight case reports, the patient characteristics were described. Seven of the eight patients were male (88 %) and the average age was 11 years (range 7–15 years).

#### Clinical presentation and physical examination

All included studies described the symptoms and findings at physical examination. In five patients, a swelling of the elbow was noted (50 %) [2, 6, 7]. Pain in the elbow was presented in five patients (63 %) [2, 6, 7, 9]. A limited range of motion at presentation was described in all seven case reports (100 %), and an extension contracture of about 15° at presentation was seen in four patients (50 %) [7–9]. In one patient, progressive bowing of the elbow was noted (13 %) [10]. Beyer et al. described a patient with a 15° flexion limitation and 20° varus deformity (13 %) [1]. In one patient, a flexion and extension limitation at presentation was noted (13 %) [6].

#### Radiological evaluation

In all reports, conventional radiography was used for diagnosing Hegemann’s disease. Irregularity of the trochlea was described in six patients (75 %) [2, 6–9] and sclerosis of the trochlea was shown in three case reports (38 %) [6, 9, 10]. Szepesi noted a fragmented trochlea (13 %) [9]. Irregularity of the epiphysis was seen in two patients (22 %) [6, 9]. Flattening of the trochlear ossific nucleus was described in one patient (11 %) [10]. Beyer et al. [1] noted osteolysis of the trochlea (11 %), and in the patient reported by Martin and Ehrenpfordt, a progressive increase of trochlea surface was seen (13 %) [7].

#### Treatment

In four case reports, the treatment for Hegemann’s disease was described. A conservative treatment was recommended [2, 6, 7, 9]. In three patients, rest was described as the treatment for Hegemann’s disease (60 %) [7, 9]. Szepesi advised less physical activities, Martin and Ehrenpfordt recommended less movement and Ito et al. advised to avoid vigorous sports activities by the epiphyseal closure and to avoid certain forms of sports involving hanging and throwing exercises [6, 7, 9].

#### Outcome (measures)

Six case reports described the outcome measurements. The average follow-up time was 32 months (range 12–16 months).

Radiography was used as an outcome measurement in all six studies [1, 2, 6, 7, 9, 10]. In one patient, a reduced size of the trochlea, with the radius being longer than the ulna at the proximal radio-ulnar joint, was shown (17 %) [6].

Clinical symptoms were used as an outcome measurement in five patients [2, 6, 7, 9]. No elbow pain after treatment was reported in four patients (80 %) [6, 7, 9], although Hegemann described a case in which the patient still had intermittent pain (17 %) [2].

In two patients, function of the elbow was used as the outcome measurement [2, 9]. In the case presented by Hegemann, a range of motion restriction still existed [2] and loss of extension of the elbow was shown in one patient [9].

### Fishtail deformity

Fifteen case reports regarding fishtail deformity including 58 patients were analysed [11–25]. The study and patient characteristics are shown in Table 3.

**Table 3:** Patients and study characteristics of patients with fishtail deformity on X-ray

Author(s), year, country	Study type	No. of males (%)	Age at injury, mean (years)	Follow-up (months)	Aetiology	Symptoms	Treatment for fishtail deformity	Outcome parameters
McDonnell and Wilson, 1948, USA [11]	Case reports	5	–	–	4× supracondylar fractures, 1× Lateral condylar fracture	–	–	X-ray
Wadsworth, 1964, England [12]	Case reports	3 (100)	6	48	Capitellum fracture	–	–	ROM, valgus deformity, X-ray
Jakob et al., 1975, Tunisia [13]	Case report	1 (100)	8	24	Lateral humeral condyle fracture	–	–	Clinical symptoms, X-ray
Rutherford, 1985, New Zealand [14]	Case reports	9	6	71	Lateral humeral condyle fracture	–	–	X-ray
Amgwerd and Sacher, 1990, Germany [18]	Case report	2	–	18	Lateral humeral condyle fracture	–	–	Valgus deformity, X-ray
Valdiserri et al., 1993, Italy [19]	Case report	1	–	72	External humeral condyle fracture	–	–	ROM, X-ray
Hasler and von Laer, 1998, Germany [20]	Case reports	8 (63)	–	120	Lateral condyle fracture	–	–	X-ray
Nwakama et al., 2000, USA [21]	Case reports	3 (100)	5	75	1× distal humeral fracture, 1× supracondylar humerus fracture, 1× medial condylar fracture	–	–	Clinical symptoms, X-ray
Skak et al., 2001, Scandinavia [15]	Case reports	2	8	84	Lateral humeral condyle fracture	–	–	X-ray
Bronfen et al., 2007, France [16]	Case reports	6 (83)	6	74	Supracondylar fracture	–	–	Clinical symptoms, valgus deformity, ROM, X-ray, loose bodies
Schulte and Ramseier, 2009, Belgium [22]	Case report	1 (0)	10	–	Supracondylar fracture	–	–	X-ray
Namba et al., 2011, Japan [23]	Case report	1 (100)	2	168	Medial condyle fracture	–	–	ROM, X-ray
Cates and Mehlman, 2012, USA [24]	Case report	1	10	36	Lateral humeral condyle fracture	–	–	Clinical symptoms, valgus deformity, ROM, X-ray
Glotzbecker et al., 2013, USA [17]	Case reports	14 (64)	10	55	6× supracondylar fracture, 4× lateral humeral condyle fracture, 3× elbow fracture, 1× unknown injury	Pain, limited ROM	7× observational if ROM 25–130° and minimal pain. Patients with or developing pain, ROM loss, daily/weekly cracking symptoms or presence of loose bodies and arthroscopic joint debridement	X-ray
Sağlam et al., 2014, Turkey [25]	Case report	1 (100)	15	52	Medial humeral condyle fracture	–	–	ROM, X-ray

*ROM* range of motion

#### Patient characteristics

In all case reports, the patient characteristics were described. The average age at injury was 7.8 years (range 2–15 years) [12–17, 21–24]. In nine studies, the sex of the patient was noted [12, 13, 16, 17, 20–23, 25]. Most patients were male (74 %). Twenty-eight patients had a lateral humeral condyle fracture (48 %) [11, 13–15, 17, 18, 20, 24], 18 patients had a supracondylar fracture (31 %) [11, 16, 17, 21, 22], three patients had a capitellum fracture (5 %) [12], three patients had a medial humeral condyle fracture (5 %) [21, 23, 25], one patient had an external humeral condyle fracture (2 %) [19], in four patients the exact location of the fracture was unknown (7 %) [17, 21] and in one patient the injury is unknown (2 %) [17]. The average follow-up time was 69 months [12–21, 23–25].

#### Clinical presentation and physical examination

In only one study was the clinical presentation and physical examination described [17]. Pain and limited range of motion was shown in 12 of the 14 patients evaluated in this study (86 %). Varus alignment was noted in five patients (range 3–8°), valgus in six patients (range 6–18°) and neutral in three patients. The average loss of extension is 12° (range 0–40°) and the average flexion loss is 11° (range 0–40°).

#### Radiological evaluation

In all studies, a radiograph was done to diagnose fishtail deformity [11–25]. Computed tomography (CT) and magnetic resonance imaging (MRI) are advised to diagnose the severity of fishtail deformity and its complications [17].

#### Treatment

Only one study described the treatment for fishtail deformity [17]. Glotzbecker et al. [17] recommended observational therapy in patients with a range of motion of 25–130° and minimal pain. In the case of progressive pain or increase in loss of range of motion, painful cracking symptoms or presence of symptomatic loose bodies, arthroscopic joint debridement was advised.

#### Outcome (measures)

In all 15 studies, radiographs are used to measure the fishtail deformity [11–25]. The average follow-up time was 69 months (range 18–168 months). Range of motion was used as the outcome measure in seven studies [12, 14, 16, 19, 21, 24, 25]. Loss of extension was seen in 12 patients (67 %) [12, 14, 16, 21, 25] and a valgus deformity was seen in seven patients (39 %) [12, 14, 16, 18, 24]. Clinical symptoms were described in 11 patients [13, 16, 21, 24]. Movement pain was seen in seven patients (64 %) [13, 16, 21], a cracking joint was shown in two patients (18 %) [16] and an arm length difference was seen in one patient (9 %) [21].

Glotzbecker et al. recommended conservative treatment for patients with a small impairment of range of motion and a symptomatic cracking of the joint. For the patients with progressive pain or increase in loss of range of motion, painful cracking symptoms or presence of symptomatic loose bodies, operative debridement was advised. In the operated patients, pain relief was seen in 85 % of the patients, and in 100 % of the patients, an increase in range of motion was noticed, with an average gain of 35° (range 25–50°) [17].

## Discussion

Reports on patients with Hegemann’s disease and fishtail deformity are very rare.

To our knowledge, this systematic review regarding Hegemann’s disease and fishtail deformity is the first to summarise current knowledge on aetiology, radiographic findings and different treatment modalities.

Studies so far report that osteochondrosis goes through stages, similar to Perthes’ disease [3]. The aetiology of Hegemann’s disease remains unclear, although traumatic events may play a role, as in five of eight cases, a contusion, fracture or chronic repetitive micro-trauma are described. It is known that traumatic events in children may sometimes be overlooked or under-reported; therefore, a traumatic origin in the other three cases may play a role as well [26].

The trochlear epiphysis ossification centre appears after 5 years of age and develops between 8 and 13 years of age in boys. The ossification centre fuses with the metaphysis of the humerus between 13 and 16 years of age [6, 27]. The trochlear blood supply comes from two end arterioles. The lateral aspect of the medial crista, the trochlear groove and the trochlear apex are relatively hypovascular. So those structures are prone to disturbances in the blood supply and the development of avascular necrosis [4]. Trochlear avascular necrosis is characterised by disturbance of growth involving the centres of ossification of the trochlea. Therefore, fishtail deformity could be a result of avascular necrosis of the vessels supplying the lateral trochlea [4, 28]. If the lateral trochlear ossification centre development is disturbed and the remaining normal physis continues to grow, the distal humerus assumes a typical V shape: the fishtail [4, 21].

Hegemann’s disease is often diagnosed by radiography months or years after trauma [7, 10, 29]. This makes Hegemann’s disease prone to confusion with a ‘fishtail deformity.’ Fishtail deformity of the elbow is an uncommon complication usually following a distal humeral fracture in childhood [4]. Usually, the term ‘Hegemann’s disease’ is used for spontaneous or idiopathic osteonecrosis of the humeral trochlea. You can question if a disease can be called Hegemann’s disease if it is not of idiopathic origin.

**Fig. 2:**
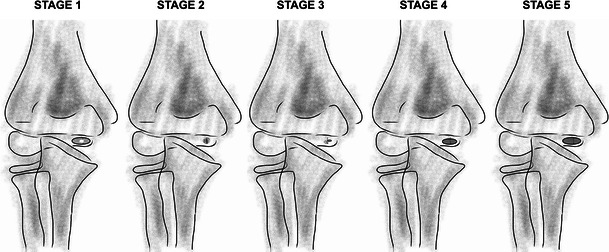
Stages of Hegemann’s disease

In all reports, standard radiographs were used for diagnosing Hegemann’s disease and fishtail deformity. There is no gold standard for diagnosing Hegemann’s disease and fishtail deformity. However, signs of fishtail deformity are shown earlier on CT and MRI, although at the time Hegemann’s disease was first diagnosed neither CT nor MRI were available. Therefore, the fishtail deformity could be another (next) stage of Hegemann’s disease, which is benign after a mild vascular disorder. A complete avascular necrosis could develop after traumatic events. Alternatively Hegemann’s disease is a benign, self-limiting stage of fishtail deformity after unrecognised injury or (repetitive) micro-trauma. Hegemann’s disease is characterised by irregularity of the trochlea and sclerosis. Schumacher et al. [30] classified Hegemann’s disease into five different stages based on radiographs: stage 1: initial loss of density and later plaque-shaped sclerosis of the centre of epiphyseal ossification; stage 2: reduction in size and condensation of the ossification centre; stage 3: loosening, accompanied by onset of new ossification; stage 4: regeneration and enlargement of the ossification centre; and stage 5: final stage (complete or partial recovery) (Fig. 2). A central deficiency of the distal humeral epiphysis is characteristic for fishtail deformity [4]. Radiographs should always be compared to the asymptomatic elbow, as the appearance of the growth plate of the trochlea differs between individuals.

Whilst Hegemann’s disease was initially presumed to be a benign condition, in four of the six cases where follow-up was done, no full recovery was seen. In early stages, rest and a total ban on all sports activities and exemption from participation in certain forms of sport involving hanging, propping and throwing exercises, together with apparatus gymnastics, have been advised until the epiphysis is closed. It is questionable if a total ban on all physical activities is mandatory in these young children; possibly, a symptom-related adjustment of activities could be enough. CT and MRI scans are helpful to confirm the presence of a premature fishtail deformity, intra-articular loose bodies and signs of osteoarthritis. If intra-articular loose bodies are found and occur with locking symptoms of the elbow, arthroscopic debridement may be indicated. Long-term follow-up studies showed that patients with fishtail deformity are probably prone to functional impairment, ongoing pain and the development of early osteoarthritis [16, 21]. It is unknown whether or not early arthroscopic debridement of the joint prevents osteoarthritis in the future.

Based on this systematic review, many aetiological aspects of Hegemann’s disease and fishtail deformity remain unclear. It is presumable that fishtail deformity is a stage of Hegemann’s disease or Hegemann’s disease is a benign stage of fishtail deformity after unrecognised injury or (repetitive) micro-trauma. Additional imaging is advised to confirm the presence of a premature fishtail deformity, intra-articular loose bodies and signs of osteoarthritis to decide if operative treatment is indicated.

There are several weaknesses in the included studies of this systematic review. The review is based on case reports, and the number of included patients in each study was low and, therefore, the strength of evidence is limited by the quality of the available studies.

Since Hegemann’s disease and fishtail deformity are very rare, higher quality studies are not likely to be performed and, thus, this systematic review provides the best level of evidence on what is known about Hegemann’s disease.

## Conclusion

Future studies on Hegemann’s disease and fishtail deformity should ideally investigate the aetiology to prevent those diseases. As long as no clear aetiology for both diseases exist and the clinical symptoms and radiographic appearance are difficult to distinguish, both entities should preferably be named a ‘vascular disturbance of the trochlear growth plate’ to overcome confusing definitions and discussions.
